# Prevalence and correlates of intimate partner violence towards female students of the University of Ibadan, Nigeria

**DOI:** 10.1186/1472-6874-14-131

**Published:** 2014-12-08

**Authors:** Joseph E Umana, Olufunmilayo I Fawole, Ikeola A Adeoye

**Affiliations:** Department of Epidemiology and Medical Statistics, Faculty of Public Health, College of Medicine, University of Ibadan, Ibadan, Nigeria; 20 Akpan Etuk street, Uyo, Akwa Ibom State Nigeria

**Keywords:** Intimate partner violence, University female students, Experience of violence, Perpetration of violence

## Abstract

**Background:**

In Nigeria, there is paucity of information on the IPV burden and experience among young women in courtship and dating relationships. This study assesses the prevalence and correlates of IPV in female undergraduate and postgraduate students in a tertiary institution.

**Methods:**

The study was a cross-sectional survey. A four-stage sampling technique was used to select 1,100 undergraduate and 255 postgraduate female students from the University of Ibadan, Nigeria. Data was collected using a 43-item self-administered structured questionnaire. Descriptive statistics and multivariate analyses were carried out at 0.05 level of significance.

**Results:**

The life-time prevalence of IPV was 42.3% (postgraduate: 34.5%, undergraduate: 44.1%; P < 0.05). Lifetime experience of psychological, physical and sexual IPV were 41.8%, 7.9% and 6.6% respectively. Recent experience (within the previous 12 months) of violence was also more frequently reported by respondents who had a previous history of physical (62.5%) (OR = 2.65; 95% CI: 2.02-3.49) and sexual (53.2%) (OR = 1.63; 95% CI:1.12-2.35) violence than respondents who had no such history. Postgraduate (OR = 0.64; 95% CI: 0.46-0.87) and married (OR = 0.53; 95% CI: 0.35-0.78) students were less likely to have experienced IPV than undergraduate and single students respectively. Students who smoked (OR = 2.46; 95% CI: 1.58-3.83); consumed alcohol (OR = 2.36; 95% CI: 1.82- 3.06); and with history of interparental violence (OR = 2.40; 95% CI: 1.88- 3.07) had a higher likelihood of experiencing violence than students who were not exposed to these behaviors. Adverse effects (such as the inability to concentrate) of IPV on academic performance were reported by 10.3% of victims.

**Conclusion:**

The prevalence of IPV was high. There is the urgent need for interventions that will reduce vulnerability by addressing modifiable risk factors like smoking and alcohol consumption. Interventions should also encourage seeking health care following violence to reduce its consequences.

**Electronic supplementary material:**

The online version of this article (doi:10.1186/1472-6874-14-131) contains supplementary material, which is available to authorized users.

## Background

Intimate Partner Violence (IPV) against women is now recognized as a problem of global magnitude, owing to its detrimental consequences on the health, social and economic welfare of women and their children [1–4]. It is a life-threatening problem primarily affecting women and girls. Exposure to IPV among women has been associated with increased morbidity and is documented as the third leading cause of mortality among women of reproductive age [2, 5]. IPV often has serious long-term consequences for the individuals involved, their families, communities, and society [6]. IPV is not restricted to married couples, but also occurs among people in courtship and dating relationships. The phenomenon cuts across all age, social and economic constellations [1–3].

However young women particularly college students experience high levels of IPV over the course of their schooling, with prevalence rates ranging between 9% and 87% [7]. Thus, it is important to understand the factors contributing to experiencing violence in intimate relationships in this high-risk population and identify ways to eliminate this behaviour.

A major constraint on the detection and prevention of IPV is the poor disclosure of abuse by women, particularly in Sub-Saharan Africa. Studies have indicated that only 1 to 4% of women disclose IPV exposure to relevant authorities due to a lack of trust, “respect” for husband and family, fear of reprisal attacks, economic dependence on the abusive partner, and concern for the safety and welfare of children [8–10]. While all this may be the case, data from the Sub-Saharan African region indicate that significant proportions of women themselves justify IPV [11, 12], providing anecdotal support for the importance of the role played by attitudes towards IPV in disclosure of and exposure to IPV.

In Nigeria, wife beating is one of the commonest (31.3%) forms of violence against women by husbands or other intimate male partners [13]. Fawole and her co-workers also reported that 24% of young women had been violated by partners and a prevalence of 30.4% for sexual violence among young female hawkers in southwestern Nigeria [14]. Similarly, Fatusi and Alatise [15] in Ile-Ife reported a sexual abuse prevalence of 19.9% in a study on women’s experiences of intimate partner violence. Although violence against women is pervasive, there are only few studies documenting the magnitude of the problem especially among female university students in Nigeria. Thus this study sought to fill this knowledge gap by determining the prevalence and identifying the correlates of IPV among students of a tertiary institution in Nigeria.

Specifically it determined the prevalence of intimate partner violence experienced by the female students, assessed their attitudes towards intimate partner violence, documented the victims’ sources of help or support and identified factors that protect or mitigate violence against women.

The ecological framework used in this study [11] viewed interpersonal violence as the outcome of interaction among many factors at different levels namely: individual, relationship, the community, and the society.

At the individual level, personal history as well as other behavioural factors that may increase the vulnerability of IPV were included. For example being a victim of child maltreatment, smoking, alcohol and/or substance abuse and a history of aggressive behaviour or abuse among the young women [11]. The relationship factors are partners’ education, smoking status, alcohol use, ethnicity, religion and history of physical fights, so we explored partners’ education, alcohol use, smoking status and history of physical fights. Community contexts in which social relationships occur in the school also influence violence. Societal factors are those factors which aggravate or inhibit the occurrence of violence in the society. These societal factors explored included the social and cultural norms such as those around male dominance over women, and cultural norms that justify violence as an acceptable method to resolve conflicts [11].

## Methods

### The study area

The study area was the University of Ibadan, the premier university in Nigeria. The campus is located in the city of Ibadan, the capital of Oyo state, in south western Nigeria. The population of students in 2005/2006 session was approximately 18,000, comprising 34% female, 35% postgraduate and 65% undergraduate.

### Study population

The study population was the female students resident in the hostels on campus. This population was irrespective of age, ethnic group, year of study, marital status, socio-economic and religious affiliations.

### Study design

The analytical cross-sectional survey design was used. The study period was from April to July, 2008.

### Sample size estimation

Sample size was estimated using Kish’s single population proportion formula for cross sectional surveys assuming the proportion of students who had experienced abuse was 31% (0.31) [13]. A minimum sample size of 1,141 was obtained.

### Sampling technique

A four step multistage sampling technique was used. In the first stage the six female hostels in the university were stratified, based on the level of study programme, into undergraduate, postgraduate and a mix of both undergraduate and postgraduate hostels. In the second stage, one hostel each from the three groups (undergraduate, postgraduate, and mixed) were selected from each stratum using simple random sampling technique. In the third stage, systematic sampling technique was used to select rooms in the hostels. The first room was randomly selected and subsequently every other third room on each floor was selected for the survey. Finally, all consenting occupants in the selected room were given the questionnaires. The students who were not available in the selected rooms at first visit were revisited until they were met and given the questionnaire. A total of 1355 students returned their questionnaires, comprising 1,100 undergraduate and 255 postgraduate students from the 1375 who received the questionnaire.

### Data collection instrument

The questionnaire was developed by adapting the questionnaire used in the WHO Multi-Country Study on Women’s Health and Domestic Violence against Women [3]. The adaptations made included rephrasing questions on knowledge, prevalence, and health consequences of IPV; and history of childhood abuse. Also questions on the impact and coping with IPV and attitude towards IPV were included. Contributions from local experts on gender-based violence and review of relevant literature was also done to ensure questionnaire was appropriate for the study area (see Additional file 1).

The questionnaire was pre-tested on 15 randomly selected female students of another tertiary institution in Ibadan. The final instrument consisted of a semi-structured self-administered questionnaire, which comprised 43 questions. The questionnaire was essentially self-administered by the respondents because of the sensitive nature of the questions. However, data collection was done in the presence of trained research assistants in order to ensure a higher response rate as well as assist respondents with necessary clarifications.

### Ethical considerations

Ethical approval for this study was obtained from the Oyo State Research Ethical Review Committee. The participants gave verbal informed consent before data collection and were free to decline or withdraw at any point during the research. In order to assure anonymity and confidentiality, information on names and other forms of identification were not included in the questionnaire. The questionnaires were filled out in private and kept safely with the research assistants. The research assistants were trained on the importance of strict confidentiality of all responses given, and on referral of students who needed care.

## Results

### Socio-demographic characteristics of respondents

As shown in Table 1, most of the respondents (74.5%) were less than 25 years. Majority were single (91.7%) and undergraduates (81.1%), with about half (53.9%) in their first to third year of study and 27.2% in their fouth to sixth year. A high proportion (85.3%) was Christian, and was sponsored by their parents (82.6%).

**Table 1:** **Respondents’ socio-demographic characteristics**

Variables	n (%)
**Age group (years)**	
<20	399 (29.4)
20-24	611 (45.1)
25-29	262 (19.3)
30-34	58 (4.3)
35+	25 (1.9)
**Marital status**	
Single	1242 (91.7)
Married	107 (7.9)
Divorced	6 (0.4)
**Study level**	
1st - 3rd year	731 (53.9)
4th - 6th year	369 (27.2)
Masters degree	218 (16.1)
Ph.D	17 (1.3)
No response	20 (1.5)
**Religion**	
Christianity	1156 (85.3)
Islam	182 (13.4)
Traditional	14 (1.1)
No response	3 (0.2)
**Sponsor**	
Mother	221 (16.3)
Father	898 (66.3)
Husband/boyfriend	97 (7.2)
Other relatives	51 (3.7)
Government/scholarship	6 (0.4)
No response	82 (6.1)

### Types of intimate partner violence experienced

**Figure 1:**
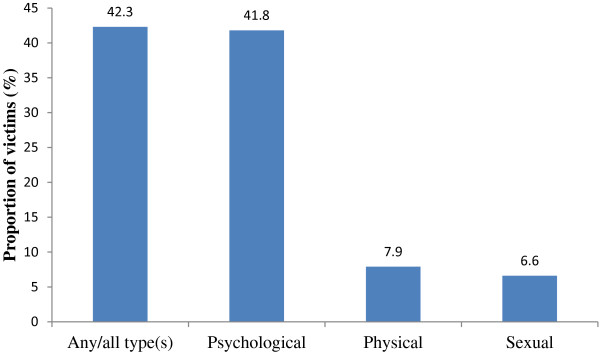
**Frequency distribution of types of intimate partner violence experienced.**

Figure 1 indicates that the lifetime prevalence of experience of any IPV was 42.3%. The proportions who had experienced psychological, physical and sexual IPV were 41.8%, 7.9%, and 6.6% respectively.

### Consequences of intimate partner violence

Injuries sustained by the victims of IPV included: cuts, punctures, bites (55.0%); scratches, abrasions, bruises (48.3%); and sprains, dislocations (18.3%). Academic performance of victims was also affected as violence caused loss of concentration (71.1%), loss of self-confidence (68.9%), and school absenteeism (56.0%) (Table 2).

**Table 2:** **Types of injuries sustained following intimate partner violence**

Characteristics	
Types of injury*	
Cuts, punctures, bites	33 (55.0)
Scratches, abrasions, bruises	29 (48.3)
Penetrating injuries, deep cuts	14 (23.3)
Sprains, dislocations	11 (18.3)
Ear injuries, eye injuries	8 (13.3)
Fractures	5 (8.3)
Broken teeth	5 (8.3)
Injuries to the genitals	5 (8.5)
No response	7 (11.7)
Effect of partner’s violent behaviour on studies*	
Unable to concentrate	407 (71.1)
Loss of self-confidence	395 (68.9)
Unable to study/school absenteeism	321 (56.0)
Skipped semester/s	22 (3.8)

*Multiple responses present.

### Experience of intimate partner violence

**Figure 2:**
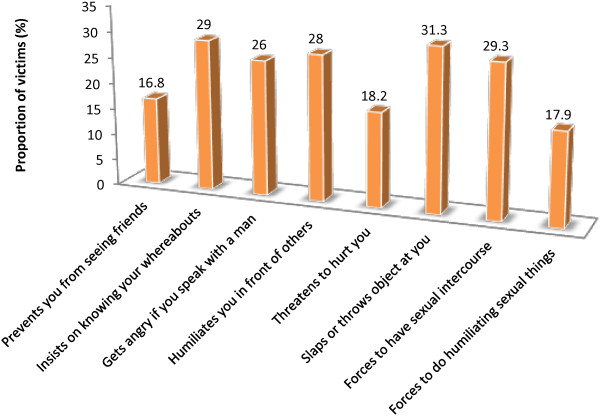
**Common intimate partner violence experience by students.**

Figure 2 shows the proportion of victimized respondents whose partners often prevented them from seeing friends (16.8%), insisted on knowing their whereabouts (29.0%), got angry if they spoke with other men (26.0%), and forced to have sexual intercourse (29.3%). Other forms of violence experienced included being slapped (31.3%), and humiliated (28.0%), among others.

### Experience of childhood abuse

From Table 3, the reported experience of childhood physical, psychological, and sexual abuse by respondents were 28.2%, 6.6%, and 9.6% respectively.

**Table 3:** **Frequency distribution of experience of childhood abuse**

Characteristics	Number (N = 1355)	Percentage (%)
Before the age of 15, did your parents or guardians ever severely hit you with a fist, kick you, or push you?	382	28.2
Before the age of 15, did anyone undress or do things to belittle you?	90	6.6
Before the age of 15, did anyone less than 5 years older than you use physical force to touch you in a sexual way?	130	9.6

### Attitude towards intimate partner violence

The respondents’ expectations, beliefs, understanding or perceptions of intimate partner violence are shown in Table 4. Some (58.8%) of the respondents believed that a good woman obeys her husband even if she disagrees with his views, family problems should be discussed within the family (67.9%), and if a man beats his wife others should interfere (41.2%). The respondents agreed (19.3%) that it is necessary for a man to show his wife/partner who the boss is in the home, and it is a woman’s obligation to have sex with her husband anytime he wants it (36.4%).

**Table 4:** **Frequency distribution of attitude towards intimate partner violence**

Characteristics	Agree	Don’t know	Disagree	Total
	n (%)	n (%)	n (%)	n (%)
A good woman obeys her husband even if she disagrees with his views	797 (58.8)	268 (19.8)	290 (21.4)	1355 (100)
Family problems should be discussed within the family	920 (67.9)	233 (17.2)	202 (14.9)	1355 (100)
It is necessary for a man to show his wife/partner who is boss at home	261 (19.3)	326 (24.0)	768 (56.7)	1355 (100)
A woman should choose her friends even if her partner disagrees	343 (25.3)	396 (29.2)	616 (45.5)	1355 (100)
It’s a woman obligation to have sex with her husband anytime he wants it	493 (36.4)	347 (25.6)	515 (38.0)	1355 (100)
If a man beats his wife others should interfere	558 (41.2)	423 (31.2)	374 (27.6)	1355 (100)

### Correlates of intimate partner violence experience

Table 5 shows that the postgraduate students had less likelihood of experiencing violence (OR = 0.64; 95% CI: 0.48-0.87), than undergraduates below the fourth year of schooling. Respondents who consumed alcohol (OR = 2.36; 95% CI: 1.82-3.06), smoked cigarettes (OR = 2.46; 95% CI: 1.58-3.83), had history of interparental violence (OR = 2.40; 95% CI: 1.88- 3.07) were significantly associated with experiencing violence than reference categories.

**Table 5:** **Logistic regression of correlates of intimate partner violence experience**

Variables	Odds ratio	Confidence interval	p-value
**Level of study**			
1st-3rd year	1.000		
4th-6th year	0.895	0.695-1.152	0.389
Postgraduate	0.644	0.479-0.866	0.004
**Smokes**			
No	1.000		
Yes	1.459	0.880-2.420	0.143
**Consumes alcohol**			
No	1.000		
Yes	2.272	1.685-3.063	<0.001
**History of interparental violence**			
No	1.000		
Yes	2.542	1.965-3.288	<0.001
**Marital status**			
Married	1.000		
Single	3.223	2.042-5.085	<0.001

## Discussion

IPV against the students was a prevalent problem and manifested as physical hurt, sexual assault and harassment, and psychological abuse. The prevalence of IPV was similar to the reported estimates in a study on IPV to female students in the United States of America. A lifetime prevalence of 42.1% was reported among college students in Philadelphia [16]. This suggests that IPV is a global phenomenon.

However, the prevalence of physical violence obtained was lower than 28% reported in the Nigeria Demographic and Health Survey [17]. The influence of women’s education on experience of physical violence was protective. The association between a woman’s education and risk of IPV can be explained through a number of mechanisms. First, education could provide a woman with knowledge and skills needed to improve her assertiveness in a relationship, thereby decreasing her likelihood of experiencing physical violence. Also, an educated partner may also be more valued and respected compared with an uneducated partner, thereby protecting her further from abuse [18].

The influence of women’s education on sexual violence was surprising. It is expected that a higher level of education should lead to an increased negotiating skills and power and that this would contribute to a lower risk of sexual violence. However, this was not the finding in this population. The prevalence of sexual violence in the students was similar to the 7% [17] reported for women 15–49 years old in the general population.

Violence in intimate relationships may start during courtship. Thus efforts to prevent dating partner violence should commence early when individuals are of school age and before behaviours become fixed. Also, acts of physical assault should not be tolerated and should be punished among young people. Unfortunately, the prevalence of IPV and the harms associated with this behaviour are often ignored, not reported or underestimated. The society tends to blame victims believing they have called for the experience or they should not be in relationships [9]. Thus dating violence within the context of an intimate relationship often goes unrecognized. Hence, most research and reporting of IPV are directed at married or cohabitating women [9, 12, 13]. Thus there is a need to broaden the scope of these studies to incorporate unmarried persons, because violence commences most frequently in early adulthood [7] and may progress to when partners are married or co-habiting. This is one of the strengths of our study.

Alcohol consumption and witnessing domestic violence as a child were correlates of IPV experience. The dis-inhibition associated with alcohol may result in diminished ability to avoid violence [19]. Alcohol abuse may result in partner neglect which may also facilitate development of tension within relationship that may result in violence. Alcohol use has also been reported to be associated with having multiple sexual partners [19], an issue that often fuels couple discord.

Students who experienced childhood physical and sexual abuse were significantly more likely to experience violence in adulthood. Individuals who experienced violence are more likely to use violence in the home than those who had experienced little or no violence. Children are not only affected by experiencing violence, they may also be impacted by observing violent incidents that occur between their parents. Researches have found that children who grow up in violent relationship tend to accept violence as the norm. Thus violence has intergenerational repercussion [20, 21].

Most victims did not seek help. This is consistent with findings in south eastern Nigeria where victims accepted violence as their lot, out of fear of being stigmatized and from self blame [9]. It has been suggested that the internalization of blame makes it difficult to seek help, report or escape, as the victim takes responsibility for ending the abuse [22].

Some students did not know how or where to get help after being violated. The few who did reported to family members. This is consistent with the African traditional norms that consider intimate relationships as a family issue and not a individual affair [9]. The implication of this is that there is need for education and creation of awareness on IPV, and acquisition skills by women to prevent IPV.

Many victims reported academic difficulties including absenteeism, interrupted studies, inability to concentrate or study and loss of self-confidence. In addition to affecting academic performance, the emotional and behavioural difficulties experienced by abused college women have a negative impact on multiple facets of their lives. These included relationship difficulties and problems with drug/alcohol abuse. These behaviours often interfere with victims career achievement and ultimately may affect their economic attainment [23, 24]. Victims of repeated violence experience more serious consequences than victims of one incident [25]. Thus university authorities will benefit from sensitization on short and long term consequences of IPV on students.

The type and frequency of injuries victims sustained could serve as an index of suspicion to health authorities to investigate the occurrence of violence among the students. Some victims were hospitalized as a result of the injuries sustained. Most of the injured did not disclose to the health worker the cause of their injuries. Many of the victims took a leave from their studies due to the violent acts. Thus health care providers of the campus health care services need to be sensitized to enable them screen and detect cases of IPV early. This is particularly important since most victims prefer to keep silent.

Only a small percentage (6.2%) of the female students justified IPV. This could be because of their high education level and general good health awareness. This is in contrast to a study among general population of Nigerian women where 66.4% and 50.4% of ever-married and unmarried women respectively agreed that a husband is justified to beat his wife, showing that domestic violence is deep-rooted in the culture and that wife beating is considered a prerogative of men [26]. Kolawole [27] showed that support for wife beating was negatively associated with education. Also Antia [28] reported that women with higher level of education had less tolerant attitudes towards IPV. Promotion of female education is a factor that parents and policy makers can promote to ensure close conjugal relationships that would minimize violence against women.

The attitude of victims of violence is crucial to the success of anti-violence intervention programmes. When victims perceive IPV to be an integral part of marriage or relationship, they are unlikely to report such incidents of violence to appropriate authorities, or to leave the relationship [29].

One of the limitations of this study was that it was not possible to determine causal relationships but only to test for associations because of the cross sectional nature of the study. It was also not possible to describe the temporal relationship between some of the factors observed to be associated with (e.g. alcohol use) experience of violence. There may have been recall bias because of the retrospective nature of some of the factors (such as child maltreatment) which were explored. Finally, some respondents may have been reluctant or under estimated experience of violence due to social desirability bias. Notwithstanding, our study provides useful information that could guide students, parents, school authorities, programme managers and policy makers on how to avoid violence in schools. Further studies that address both female and male students and obtain information on perpetration of violence are however recommended.

## Conclusions

The prevalence of IPV was high and suggests that primary and secondary prevention of IPV in tertiary institutions is urgently required. Multi-pronged interventions that focus on changing IPV norms, conflict-management skills, and alter the help-seeking behaviour of victims are also essential among students. University authorities need to implement strategies that encourage reporting of IPV and ensure that such reports are managed appropriately.

## Authors’ information

JEU, OIF, and IAA are all citizens of Nigeria.

## Electronic supplementary material

Additional file 1: **Questionnaire.** (PDF 201 KB)
