# Supplementary material for: Prevalence and correlates of intimate partner violence towards female students of the University of Ibadan, Nigeria
Source: BMC Womens Health. 2014 Dec 8;14:131. doi: 10.1186/1472-6874-14-131 (PMC4295485; doi:10.1186/1472-6874-14-131)
Supplement: Supplementary file 1 — Additional file 1: Questionnaire. (PDF 201 KB) [file 12905_2012_504_MOESM1_ESM.pdf]

**PREVALENCE AND DETERMINANTS OF INTIMATE PARTNER VIOLENCE TOWARDS FEMALE STUDENTS OF THE UNIVERSITY OF IBADAN, IBADAN.**

Hello, my name is Umana Joe. I am a postgraduate student conducting a survey in the University of Ibadan to learn about female student's health and life experiences. I want to assure you that all of your answers will be kept strictly secret. No record of your name or address will be kept. You have the right to stop your participation at any time, or skip any questions that you don't want to answer. Thank you for your anticipated co-operation in answering the questions honestly. Your experiences could be very helpful to other women in Nigeria.

**SECTION 1: SOCIO-DEMOGRAPHIC DATA**

**1.Your age in years (as at your last birthday).....................**

**2.Marital status**

1.[ ] Single, never married 2.[ ] Married 3[ ] Separated

4.[ ] Widowed 5.[ ] Divorced

**3. What is your level of study in this university?**

1.[ ] Undergraduate (1st year-3rd year) 2.[ ] Undergraduate (1st year-3rd year)

3.[ ] Masters degree programme 4.[ ] Ph.d programme

5.[ ] Others(specify)..............................................

**4. Who pays your school fees?**

1.[ ] Mother 2.[ ] Father 3.[ ] Husband/boyfriend 4.[ ]Relatives

5.[ ] Govt/scholarship 6.[ ] Others (specify)

**5. Tribe**

1.[ ] Hausa 2.[ ] Yoruba 3.[ ] Ibo 4.[ ] Others (specify)

**6. Religion**

1.[ ] Christianity 2.[ ] Islam 3.[ ] Traditional 4.[ ] Others (specify)

**7. Do you smoke cigarettes?**

1.[ ] Yes 2.[ ] No

**8. If yes to question No. 7, on the average how many sticks per day?**

1.[ ] 1-5 2.[ ] 6-10 3.[ ] 11-20 4.[ ] 20 and above

**9. Do you take alcoholic drinks?**

1.[ ] Yes 2.[ ] No

**10. If yes to question No. 9 on the average what is your intake?**

1.[ ] Everyday or nearly everyday 2.[ ] Once or twice a week 3.[ ] 1-3 times a month

4.[ ] Occasionally, less than once a month 5.[ ] Other (specify)...........................................

**11. Do you occasionally take drugs ( e.g. marijuana, cocaine, heroine etc)**

1. [ ] Yes 2. [ ] No

**12. Did you as a child sometimes see your parents quarelling or fighting at home**

1. [ ] Yes 2. [ ] No

If you are married or have a boyfriend or a (sexual) partner, please answer questions 13 to 20, otherwise proceed to question 21.

**13. Your husband's/ partner's/ boyfriend's age in years ( as at his last birthday)...................**

**14. Does your husband/ partner/ boyfriend smoke cigarette?**

1.[ ] Never 2.[ ] 1-5 sticks of cigarette per day 3.[ ] 6-10 sticks of cigarette per day

4.[ ] 11-20 sticks of cigarette per day 5.[ ] 20 and above sticks of cigarette per day

**15.Does your husband/ partner/ boyfriend take drinks containing alcohol?**

1.[ ] Never 2. [ ] Everyday or nearly everyday 3.[ ] Once or twice a week 4.[ ] 1-3 times a month 5.[ ]Occasionally, less than once a month 6.[ ] Other (specify).......................

**16. What is his educational status?**

1. [ ] None 2. [ ] primary education 3. [ ] Secondary education 4. [ ] Tertiary education

**17. What kind of work does your husband/ partner/ boyfriend do? ........................................**

**18. What is his ethnic group?**

1. [ ] Hausa 2. [ ] Yoruba 3. [ ] Igbo

**19. What is his religion?**

1. [ ] Christianity 2. [ ] Islam 3. [ ] Traditional 4. [ ] Other (Specify)........................

**20. Since you met your husband/ partner/ boyfriend, has he been invioled in a physical fight with another man?**

1. [ ] Yes 2. [ ] No 3. [ ] Don't know/ don't remember

**SECTION 2: KNOWLEDGE ABOUT INTIMATE PARTNER VIOLENCE**

**21. In your opinion, which of the following actions by a man would you consider as intimate partner violence?**

| | | YES | NO |
|---|---|---|---|
| a | Tries to keep his wife/ girlfriend from seeing her friends | | |
| b | Tries to restrict contact with her family of birth | | |
| c | Insists on knowing where his wife/ girlfriend is at all times | | |
| d | Ignores his wife/ girlfriend and treats her indifferently | | |
| e | Gets angry if she speaks with another man | | |
| f | Is often suspicious that his wife/ girlfriend is unfaithful | | |
| g | Expects you to ask his permission before seeking healthcare for his wife/ girlfriend | | |
| h | Insulted his wife/ girlfriend or made her feel bad about herself | | |
| i | Belittle his wife/ girlfriend, humiliate her infront of other people | | |
| j | Done things to scare or intimidate his wife/ girlfriend on purpose (e.g. the way he looked at her, by yelling and smashing things) | | |
| k | Threatening to hurt her or someone she cares about | | |
| l | Slapped her or threw something at her that could hurt her | | |
| m | Pushed her or shoved or pulled her hair | | |
| n | Hit her with his fist or with some object that could hurt her | | |
| o | Kicked her, dragged her or beat her up | | |
| p | Threatened to use or actually used a gun, knife or other weapon against her | | |
| q | Physically force her to have sexual intercourse | | |
| r | Choke or burn her on purpose | | |
| s | Deny her money in order to hurt her | | |

**SECTION 3: PREVALENCE OF INTIMATE PARTNER VIOLENCE**

Please you are required to tick yes or no to question 22 and if you tick yes, also indicate by ticking the number of times that the action occured within the last 12 months.

**22. Has your husband/partner ever done the following to you?**

| | YES | NO | If yes, how many times in the last 12 months did he do it? | | |
|---|---|---|---|---|---|
| | | | Once or twice | A few(3-5) times | Many(more than 5) times |
| a.tried to keep you from seeing your friends | | | | | |
| b.Tried to restrict contact with your family of birth | | | | | |
| c.Insisted on knowing where you are at all times | | | | | |
| d.Ignored you and treated you indifferently | | | | | |
| e.Gets angry if you speak with another man | | | | | |
| f.Is often suspicious that you are unfaithful | | | | | |
| g.Expected you to ask his permission before seeking health care for yourself | | | | | |
| h.Insulted you or made you feel bad about yourself | | | | | |
| i.Belittled you/ humiliated you in front of other people | | | | | |
| j.Did things to scare or intimidate you on purpose (e.g. by the way he looks at you, by yelling and smashing things) | | | | | |
| k.Threatened to hurt you or someone you care about | | | | | |
| l.Slapped you or threw something at you that could hurt you | | | | | |
| m.Pushed you or shoved or pulls your hair | | | | | |
| n.Hits you with his fist or with some object that could hurt you | | | | | |
| o.Kicked you, dragged you or beat you up | | | | | |
| p.Threatened to use or actually used a gun, knife or other weapon against you | | | | | |
| q.Physically forced you to have sexual intercourse when you did not want to | | | | | |
| r.Choked or burned you on purpose | | | | | |
| s.Denied you money or other material things in order to hurt you | | | | | |
| t.Had sexual intercourse with him because you were afraid of what he might do to you | | | | | |
| u.Forced you to do something sexually that you found degrading or humiliating | | | | | |
| v.Refused to have sex with you in order to hurt you | | | | | |

**23. Since the age of 15 years, has any male other than your husband/partner/boyfriend ever beaten you or physically mistreated you in any way?**

1. [ ] Yes 2. [ ] No

**24. If yes to question 23, who did this to you? (tick the boxes that correspond to your answer and indicate the number of times it occured). If no to question 23, skip question 24 please.**

| | YES | NO | Once or twice | A few(3-5) times | Many(more than 5) times |
|---|---|---|---|---|---|
| a.Father | | | | | |
| b.Step father | | | | | |
| c. Other male family member | | | | | |
| d. Male teacher/ lecturer | | | | | |
| e. Male police/ soldier | | | | | |
| f. Male friend of family | | | | | |
| g. Stranger | | | | | |
| h. Someone at work | | | | | |
| i.Priest/Male religious leader | | | | | |
| j. Other (specify) | | | | | |

**25. Since the age 15 years, has any male other than your husband/partner/boyfriend ever forced you to have sex or to perform a sexual act when you did not want to?**

1. [ ] Yes  2. [ ] No

**24. If yes to question 25, who did this to you? (tick the boxes that correspond to your answer and indicate the number of times it occured). If no to question 25, skip question 26 please.**

| | YES | NO | Once or twice | A few(3-5) times | Many(more than 5) times |
|---|---|---|---|---|---|
| a.Father | | | | | |
| b.Step father | | | | | |
| c. Other male family member | | | | | |
| d. Male teacher/ lecturer | | | | | |
| e. Male police/ soldier | | | | | |
| f. Male friend of family | | | | | |
| g. Stranger | | | | | |
| h. Someone at work | | | | | |
| i.Priest/Male religious leader | | | | | |
| j. Other (speciify) | | | | | |

## SECTION 4: HISTORY OF CHILDHOOD ABUSE

Please you are required to answer yes or no to question 27, and if yes also indicate the number of times it occured.

**27. Before the age of 15 years, did you experience any of the following?**

| | YES | NO | If yes, how many times did it occur before your 15th birthday | | |
|---|---|---|---|---|---|
| | | | Once or twice | A few(3-5) times | Many(more than 5) times |
| a.Were you severely beaten by your parents or guardian? | | | | | |
| b.Did any man naked himself or masturbate before you? | | | | | |
| c. Did any man fondle you or have sexual penetration with you? | | | | | |

## SECTION 5: ATTITUDE TOWARDS INTIMATE PARTNER VIOLENCE

People have different ideas about family life and what is acceptable behaviour for a man and a woman in the home. I would want to know whether you generally agree or disagree with the following statements.

| | Strongly agree | Agree | Don't know | Strongly disagree |
|---|---|---|---|---|
| a.A good woman obeys her husband/intimate partner even if she disagrees with his views | | | | |
| b.Family problems should only be discussed with people in the family | | | | |
| c. If is necessary for a man to show his wife/partner who the boss is in the home | | | | |
| d. A woman should be able to choose her own friends even if her husband/partner disapproves | | | | |
| e. It is a woman's obligation to have sex with her husband/ partner anytime he wants it | | | | |
| f. If a man beats his wife, others should intefere | | | | |

**29. In your opinion, do you feel a man has a good reason to beat his wife if...**

| | Yes | No | Don't know |
|---|---|---|---|
| a.She does not complete her household work to his satisfaction | | | |
| b.She disobeys him | | | |
| c.She refuses to have sexual intercourse with him | | | |
| d.She asks him whether he has other girlfriends | | | |
| e.He suspects that she is unfaithful | | | |
| f.He finds out that she has been unfaithful | | | |
| g.She does not feel like having sex at the time | | | |
| h.He is drunk | | | |
| i.She is sick | | | |
| j.He maltreats her | | | |

## SECTION 6: HEALTH CONSEQUENCES OF INTIMATE PARTNER VIOLENCE

Health consequences refers to physical harm to the body such as cuts, burns, sprains, broken bones or broken teeth etc.

**31.Have you ever been injured as a result of physical violence by your husband/ partner?**

1. [ ] Yes 2. [ ] No 3. [ ] Don't know

**32.If yes to question No 31, how many times in your life have you been injured by (any of) your husband or partner. If no to question No 31, proceed to question No. 36**

1.[ ] Once/ twice 2.[ ] Several (3-5) times 3.[ ] More than 5 times
4.[ ] Don't know/ don't remember

**33.What type of injury did you have?**

| | | Yes | No |
|---|---|---|---|
| a) | Cuts, punctures, bites | [ ] | [ ] |
| b) | Scratch, abrasion, bruises | [ ] | [ ] |
| c) | Sprains, dislocation | [ ] | [ ] |
| d) | Burns | [ ] | [ ] |
| e) | Penetrating injury, deep cut | [ ] | [ ] |
| f) | Broken ear drum, eye injuries | [ ] | [ ] |
| g) | Fractures, broken bones | [ ] | [ ] |
| h) | Broken teeth | [ ] | [ ] |
| i) | Internal injuries | [ ] | [ ] |

j) Other (specify)..........................................................................................................

**34.Have you ever had to spend night(s) in a hospital due to injuries from your husband/ partner?**

1. [ ] Yes 2. [ ] No

**35.Did you tell the health worker the real cause of your injury?**

1. [ ] Yes 2. [ ] No

## SECTION 7: IMPACT AND COPING WITH INTIMATE PARTNER VIOLENCE

**36. Are there particular situations that tend to make your husband/ intimate partner to be violent?**

| | Yes | No |
|---|---|---|
| a. No particular reason | [ ] | [ ] |
| b. When he is drunk | [ ] | [ ] |
| c. When he has money problem | [ ] | [ ] |
| d. When he has difficulties at work | [ ] | [ ] |
| e. When he is unemployed | [ ] | [ ] |
| f. When there is no food at home | [ ] | [ ] |
| g. When there is problem with his or your families | [ ] | [ ] |
| h. When you are pregnant | [ ] | [ ] |
| i. When he is jealous of you | [ ] | [ ] |
| j. When you refuse him sex | [ ] | [ ] |
| k. When you are disobedient | [ ] | [ ] |

**37. Would you say that your husband/ partner's behaviour has affected your physical and/ or mental health?**

1.[ ] No effect 2.[ ] A little 3.[ ] A lot

38. In what way if any has your husband/ partner's violent behaviour affected your studies?

1.[ ] Studies not affected 2.[ ] Partner interrupted my studies 3.[ ] Unable to concentrate

4.[ ] Unable to study/ sick leave 5.[ ] Lost self confidence 6.[ ] Others (specify)................

**39. Did you ever go to any of the following for help?**

| | Yes | No |
|---|---|---|
| a. Police | [ ] | [ ] |
| b. Hospital or health centre | [ ] | [ ] |
| c. Social service/ shelter | [ ] | [ ] |
| d. Legal advice centre/ court | [ ] | [ ] |
| e. Traditional/ local leader | [ ] | [ ] |
| f. Women's organization | [ ] | [ ] |
| g. Priest/ religious leader | [ ] | [ ] |
| h. Others (specify) | [ ] | [ ] |
| i. Did not go for help | [ ] | [ ] |

**40. If you went for help, what were the reason that made you to go for help?**

1.[ ] Encouraged by friends/ family 2.[ ] Could not endure anymore 3.[ ] Badly injured

4.[ ] He threatened or tried to kill me 5.[ ] Thrown out of the home 6.[ ] Other (specify)......

**41. What was the main reason why you left your husband/ partner?**

1.[ ] Never left him 2.[ ] Encouraged by friends/ family 3.[ ] Could not endure anymore

4.[ ] Badly injured 5.[ ] He threatened or tried to kill me 6.[ ] Thrown out of the home

7.[ ] Other (specify)

**42. If you continued staying with your husband/ partner despite his behaviour, what were the reasons that made you stay?**

1.[ ] Did not want to leave the children 2.[ ] Sanctity of marriage

3.[ ] Didn't want to bring shame to the family 4.[ ] I love him

5.[ ] My family said I should stay 6.[ ] I forgave him

7.[ ] He threatened me/ the children 8.[ ] Nowhere to go

9.[ ] Didn't want to be single 10.[ ] Thought he would change

11.[ ] Violence normal/ not serious

43. What are your suggestions or recommendations for stopping intimate partner violence?. .
.......................................................................................................................................
.......................................................................................................................................

**THANK YOU VERY MUCH FOR YOUR TIME AND COOPERATION.**
